# Supplementary material for: Down-Regulation of Double C2 Domain Alpha Promotes the Formation of Hyperplastic Nerve Fibers in Aganglionic Segments of Hirschsprung’s Disease
Source: Int J Mol Sci. 2022 Sep 6;23(18):10204. doi: 10.3390/ijms231810204 (PMC9499397; doi:10.3390/ijms231810204)
Supplement: Supplementary file 1 [file ijms-23-10204-s001.zip › ijms-1876106-supplementary.pdf]

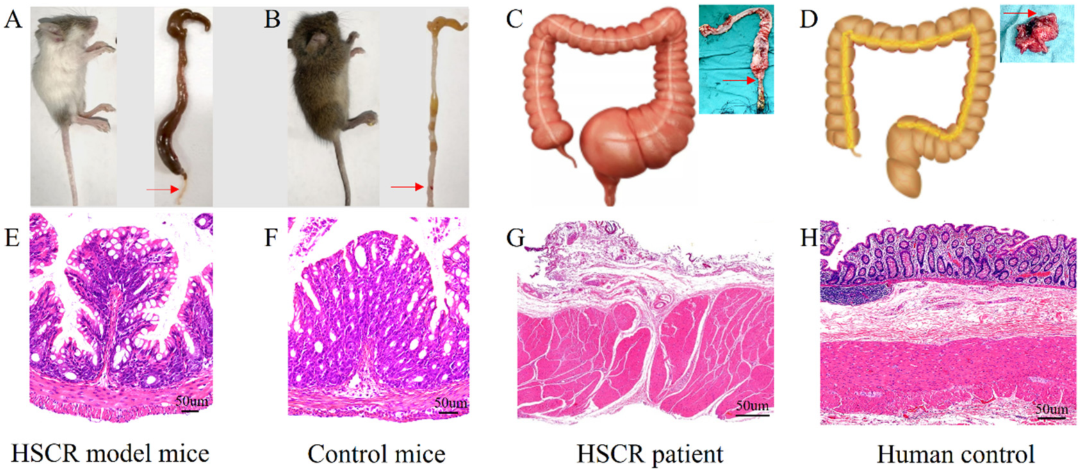

Figure S1. Morphology and pathology of colon in HSCR model mice and patients (**A–H**), comparation of morphology and pathology between HSCR and normal control colons.

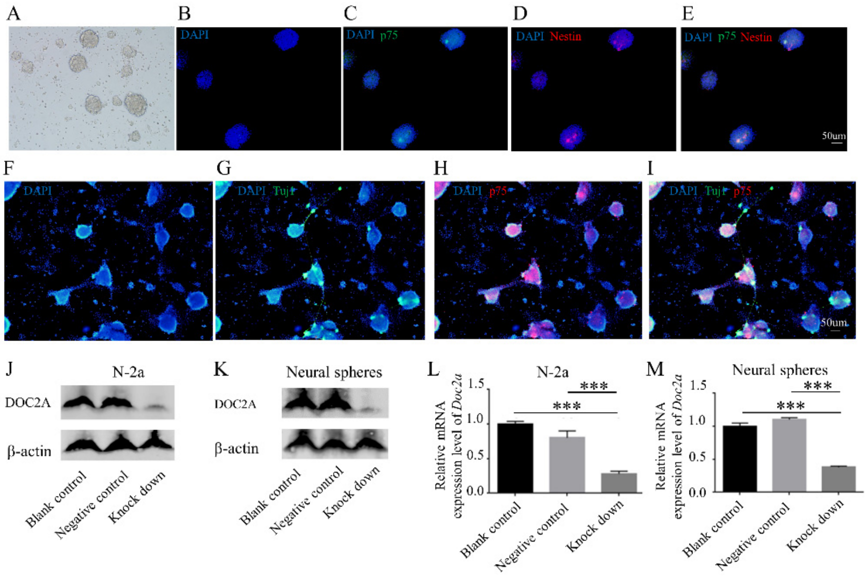

Figure S2. Identification of neural spheres, neural spheres' nerve fiber connection and successful knocking down of DOC2A in N-2a cell lines and neural spheres. (**A–E**), identification of neural spheres by double-labelling immunofluorescence (p75 and Nestin); (**F–I**), identification of neural spheres' nerve fiber connections by double-labelling immunofluorescence (p75 and Tuj1); (**J–M**), identification of successful knocking down of DOC2A in N-2a cell lines and neural spheres. ***, $p < 0.001$, one-way ANOVA with Bonferroni post-hoc, N = 3.

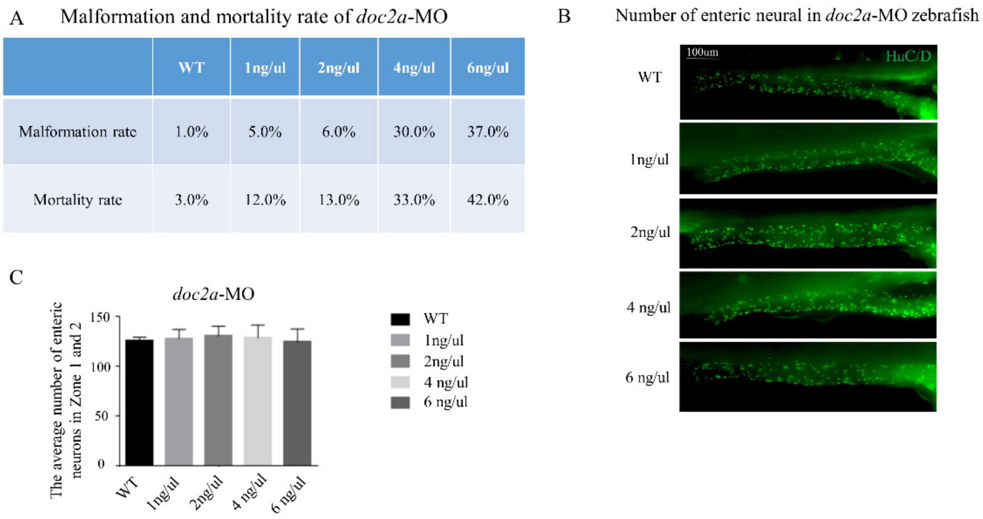

	WT	1ng/ul	2ng/ul	4ng/ul	6ng/ul
Malformation rate	1.0%	5.0%	6.0%	30.0%	37.0%
Mortality rate	3.0%	12.0%	13.0%	33.0%	42.0%

Figure S3. The phenotype of enteric neuron in *ret*-MO zebrafish.
(**A**), Malformation and mortality rates of *ret*-MO; (**B**), The number of enteric neurons in *ret*-MO zebrafish; (**C**), The average number of enteric neurons in Zone 1 and 2.

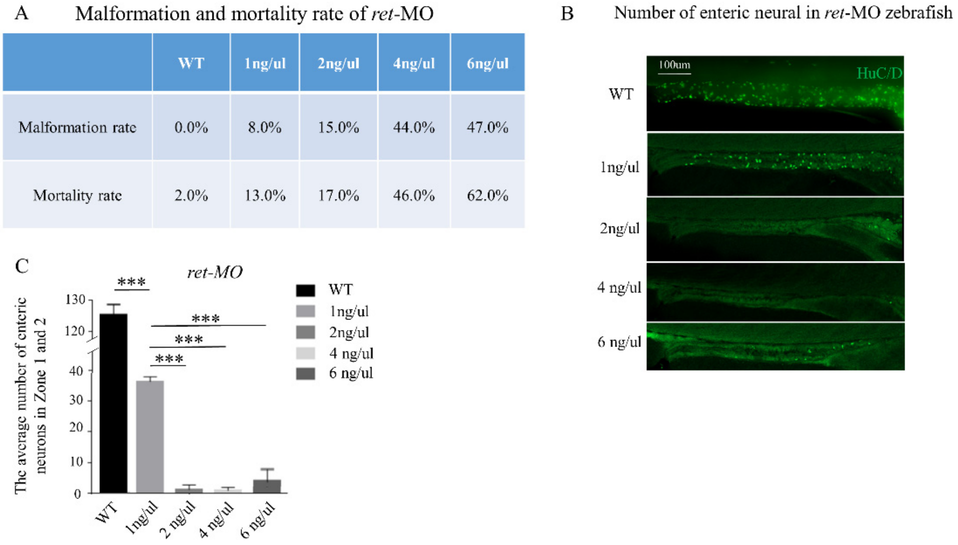

	WT	1ng/ul	2ng/ul	4ng/ul	6ng/ul
Malformation rate	0.0%	8.0%	15.0%	44.0%	47.0%
Mortality rate	2.0%	13.0%	17.0%	46.0%	62.0%

Figure S4. The phenotype of enteric neuron in *doc2a*-MO zebrafish.
(**A**), Malformation and mortality rates of *doc2a*-MO; (**B**), The number of enteric neurons in *doc2a*-MO zebrafish; (**C**), The average number of enteric neurons in Zone 1 and 2. ***, $p < 0.001$.
